# Neurological Soft Signs in Patients with Schizophrenia May be Related to Treatment Resistance

**DOI:** 10.1192/j.eurpsy.2025.624

**Published:** 2025-08-26

**Authors:** S. Elmas, A. Üçok

**Affiliations:** 1 Psychiatry, Kahta State Hospital, Adıyaman; 2 Psychiatry, Istanbul University Istanbul Faculty of Medicine, Istanbul, Türkiye

## Abstract

**Introduction:**

Motor abnormalities in schizophrenia are important as they are measurable objective parameters. Neurological soft signs (NSS), which are one of the primary motor abnormalities, are minor motor and sensory abnormalities that cannot be localised to a specific brain region, reflect the areas of sensory integration, motor coordination and motor sequencing. Instrumental measures of motor abnormalities have the potential to improve detection, early intervention and treatment strategies in psychotic disorders.

**Objectives:**

Our aim in this cross-sectional study was to compare NSS in patients with treatment-resistant schizophrenia (TRS) and treatment responsive schizophrenia (non-TRS) and healthy controls (HC), and to examine the relationship of NSS with sociodemographic and clinical variables, premorbid adjustment, functioning, negative and cognitive symptoms.

**Methods:**

30 TRS patients, 30 non-TRS patients and 30 HC were included in the study between November 2021 and November 2022. The inclusion criteria for the TRS group were to meet the criteria for resistance to antipsychotic treatment. Neurological Evaluation Scale (NES) was applied to all participants to evaluate NSS. The scale assesses impairment in four different functional areas: “sensory integration”, “motor coordination”, motor sequencing” and “other signs”. Brief Psychiatric Rating Scale (BPRS), Brief Negative Symptom Scale, General Assessment of Functioning (GAF), Clinical Global Impression-Severity Scale (CGI-S), Premorbid Adjustment Scale-Childhood (PAS), and a cognitive battery were applied to patients with schizophrenia. The authors declare that all methods used in this study adhere to the ethical guidelines of the corresponding national and institutional review boards for human research and are in alignment with the 1975 Helsinki Declaration, as updated in 2008. All participants provided written informed consent to participate in this study.

**Results:**

We found significant differences in the NES-Total score and all subscale scores between patients with schizophrenia and HC (all comparisons p<0,001). NSS in patients with schizophrenia were found to be higher than in HC. TRS had higher NES-Total (p=0,002), NES-Sensory Integration (p=0,001), and NES-Other Signs (p<0,001) scores than non-TRS. NES-Total score was negatively correlated with GAF score and positively correlated with BPRS, CGI-S and PAS-childhood scores. Poor performance on cognitive tests was associated with more NSS. Only the sensory integration deficits were found to be associated with negative symptoms.

**Conclusions:**

Our findings suggest that NSS examination in patients with schizophrenia may indicate treatment resistance or response. NSS are associated with severity of illness, lower functioning, poor premorbid adjustment, and poor cognitive performance. Longitudinal studies involving larger samples are needed to understand the course of NSS at different stages of the illness.

**Disclosure of Interest:**

None Declared

